# On-chip integratable all-optical quantizer using strong cross-phase modulation in a silicon-organic hybrid slot waveguide

**DOI:** 10.1038/srep19528

**Published:** 2016-01-18

**Authors:** Zhe Kang, Jinhui Yuan, Xianting Zhang, Xinzhu Sang, Kuiru Wang, Qiang Wu, Binbin Yan, Feng Li, Xian Zhou, Kangping Zhong, Guiyao Zhou, Chongxiu Yu, Gerald Farrell, Chao Lu, Hwa Yaw Tam, P. K. A. Wai

**Affiliations:** 1 State Key Laboratory of Information Photonics and Optical Communications, Beijing University of Posts and Telecommunications, P.O. Box72 (BUPT), Beijing, China; 2 Photonics Research Centre, Department of Electronic and Information Engineering, The Hong Kong Polytechnic University, Hung Hom, Kowloon, Hong Kong; 3 3Department of Physics and Electrical Engineering, Northumbria University, Newcastle upon Tyne, NE1 8ST, United Kingdom; 4 Guangdong Provincial Key Laboratory of Nanophotonic Functional Materials and Devices, South China Normal University, 510006 Guangzhou, China; 5 Photonics Research Centre, Dublin Institute of Technology, Kevin Street, Dublin, Ireland

## Abstract

High performance all-optical quantizer based on silicon waveguide is believed to have significant applications in photonic integratable optical communication links, optical interconnection networks, and real-time signal processing systems. In this paper, we propose an integratable all-optical quantizer for on-chip and low power consumption all-optical analog-to-digital converters. The quantization is realized by the strong cross-phase modulation and interference in a silicon-organic hybrid (SOH) slot waveguide based Mach-Zehnder interferometer. By carefully designing the dimension of the SOH waveguide, large nonlinear coefficients up to 16,000 and 18,069 W^−1^/m for the pump and probe signals can be obtained respectively, along with a low pulse walk-off parameter of 66.7 fs/mm, and all-normal dispersion in the wavelength regime considered. Simulation results show that the phase shift of the probe signal can reach 8π at a low pump pulse peak power of 206 mW and propagation length of 5 mm such that a 4-bit all-optical quantizer can be realized. The corresponding signal-to-noise ratio is 23.42 dB and effective number of bit is 3.89-bit.

The tremendous development of Internet technologies and IP business led to the ever-increasing demand in communication bandwidth. Analog-to-digital converters (ADCs) are the “bridge” that connects the real world with modern day digital technology, which plays a key role in ultra-wideband digital communication networks and information processing systems. High performance all-optical ADCs are expected to overcome the electronic bottleneck of conventional electrical ADCs and the bandwidth limitation of photonic-assisted ADCs^1^,^2^,^3^. Also, since future broadband digital systems would be supported by massively parallel processing, the photonic integration ability of all-optical ADCs is critical in meeting the requirements of on-chip mass fabrication, low power consumption, and low cost. However, the research of integratable all-optical ADCs is still at the early stage and much more work needed to be done. As the key process of all-optical ADCs, optical quantization techniques have been extensively studied in the last decade. The reported all-optical quantization schemes can be roughly divided into two categories, they are electro-optic modulators based phase-shifted optical quantization (PSOQ)^4^,^5^ and third-order nonlinear optical effects based optical quantization schemes. The former however are not strictly all-optical owing to the active operation of electro-optic modulators. By contrast, because of the totally passive operation and ultrafast response time at femtosecond level, the latter schemes show great advantages in analog bandwidth, sampling rate, and achievable quantization resolution. Typical nonlinear optical quantization schemes reported are realized in silica highly nonlinear fibers (HNLF), e.g. the spectral quantization using supercontinuum (SC)^6^ and soliton self-frequency shift (SSFS)^7^,^8^,^9^, and the multi-period phase quantization using cross-phase modulation (XPM)^10^,^11^,^12^. Compared with the SC- and SSFS-based optical quantization, the XPM-based schemes have much lower power thresholds, higher linearity of power-to-phase conversion, and better cascade ability to achieve a higher resolution^12^. However, because of the weak nonlinear interaction in the SiO_2_-based materials, high pulse power, large pulse width, and long fiber length are required. Thus, fiber-based quantization schemes cannot meet the requirements of photonic integration and low power consumption of future all-optical ADCs. Silicon-on-insulator (SOI) technology is compatible with complementary metal oxide semiconductor (CMOS) process and can confine the optical waves into submicron region because of the large refractive index difference between the silicon and substrate material^13^,^14^. But, conventional SOI waveguides suffer from inherent nonlinear absorption losses, i.e. two-photon absorption (TPA) and free carrier absorption (FCA), which significantly reduce the efficiency of the nonlinear interaction. Moreover, the pulse repetition rate is greatly limited by the free carriers. The reverse-biased p-i-n diode structure should be used to eliminate the impacts of free carriers^15^. Some nonlinear signal processing schemes using XPM effect in conventional SOI waveguide have been reported, e.g. nonlinear phase modulation^16^,^17^, all-optical switching^18^, wavelength conversion^19^,^20^,^21^, and modulation format conversion^22^,^23^. However, to the best of our knowledge, there is no such report on all-optical ADCs because of the demanding power requirement and large nonlinear absorption of conventional SOI waveguides. A slot mechanism is proposed based on conventional SOI waveguide, which can provide strong sub-wavelength mode confinement in a low index nonlinear material sandwiched between two silicon wires^24^,^25^,^26^. The horizontal slot structure can minimize the scattering loss induced by the sidewall roughness, and allows unlimited slot thickness when compared to the vertical structure^27^,^28^. By filling the slot with high nonlinear index materials, e.g. the poly [bis (p-toluene sulfonate) of 2, 4-hexadiyne-l, 6-diol (PTS) single crystal polymer material (*n*_2_ = 2.2 × 10^−16 ^m^2^W^−1^ around 1600 nm), the nonlinear coefficient can be more than 2 × 10^4 ^W^−1^/m^24^,^29^. Such a strong nonlinearity enables efficient nonlinear interaction in a short propagation length of only several millimeters and low power level. Meanwhile, PTS exhibits negligible TPA and four-photon absorption (4PA), and no FCA in the near-infrared region^30^. Only three-photon absorption (3PA) at low level should be considered. Therefore, the XPM effect in the PTS based silicon-organic hybrid (SOH) waveguide is ideal for on-chip integratable all-optical quantization with large analog bandwidth, high repetition rate, and low power consumption.

In this paper, we propose for the first time an integratable all-optical quantizer using strong XPM and interference in a SOH slot waveguide based Mach-Zehnder interferometer (MZI). The dimensions of the SOH slot waveguide is carefully designed to simultaneously achieve large nonlinear coefficients of pump and probe signals and the appropriate dispersion characteristics, which ensure high efficiency of XPM effect. The nonlinear interaction and the quantization performances are numerically studied, and related problems are also discussed.

## Results

### Principle of operation

**Figure 1:**
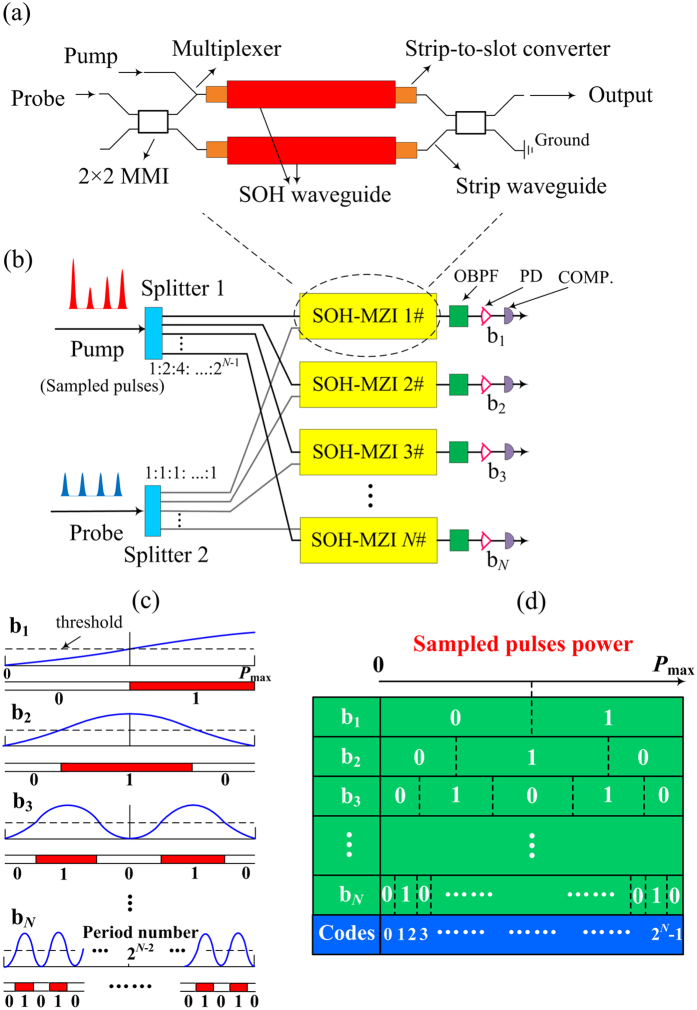
(**a**) A schematic diagram of an SOH-MZI, (**b**) a schematic diagram of the proposed all-optical quantizer with *N* quantization channels b_1_, b_2_, …, b_*N*_, (**c**) the power transfer function of the detected probe signals at the *N* quantization channels, and (**d**) the corresponding coding table. OBPF: Optical band-pass filter, PD: Photodiode, COMP: Comparator, MMI: Multimode interferometer.

The objective is to design an integratable optical quantizer which can divide the power range from 0 to *P*_max_ into 2^*N*^ equal levels, where *P*_max_ is the maximum peak power of the sampled pulses, and then to represent the 2^*N*^ power levels by using an *N* bit binary code. The physical implementation is by using the power variation of a probe signal induced by XPM in an SOH-MZI. Figure 1(a) shows the schematic of an SOH-MZI, which consists of a 2 × 2 multimode interferometer (MMI), a multiplexer, four strip-to-slot converters, two SOH waveguides, and the access strip waveguides. The MMI is used for the coupling and interference at the input and output ports of the MZI. The strip-to-slot converter is used to couple the access strip waveguides to the SOH waveguides. Coupling between the pump and probe pulses is realized by the multiplexer. A similar structure has been successfully fabricated and demonstrated in the literature^31^. A pump pulse is input into one arm while a probe pulse synchronized to the pump pulse is coupled to both arms of the SOH-MZI. The phase shift of the probe pulse inside the SOH-MZI waveguide consists of the linear phase shift induced by dispersion and the nonlinear phase shift induced by self-phase modulation (SPM) and XPM. Since by design XPM occurs in only one arm of the SOH-MZI, the probe pulses in the upper and lower arms will have a phase difference. When combined at the end of the interferometer, the output power of the probe varies periodically with the input power of the pump pulses to the SOH-MZI because of the phase difference. The output power of the probe from the SOH-MZI is given by^32^


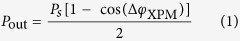


where *P*_s_ is the probe pulse peak power, ∆φ_XPM_ is the XPM-induced phase shift from the pump to probe pulse. The linear phase shift and SPM-induced phase shift of the probes in two arms are identical by design so that they are not appear in Eq. (1). The phase difference ∆φ_XPM_ can be roughly estimated by 2γ_sp_*P*_p_*L*_eff_, where γ_sp_ is the nonlinear coefficient, *P*_p_ is the peak power of the pump pulses entering the SOH waveguide, and *L*_eff_ is the effective propagation length of the waveguide. Thus, the XPM-induced phase shift is directly proportional to the pump pulse peak power, i.e. the peak power of the sampled pulses, which can be used to realize the quantization process.

Figure 1(b) shows the schematic of the proposed *N*-bit quantizer. Sampled pulses at different peak power levels, which can be realized by four-wave mixing (FWM) in silicon waveguides, are used as the pump signal. The probe signal at the same peak power level is generated at the appropriate wavelength by a mode-locked laser diode (MLLD) and kept synchronous with the pump. After splitting inside Splitter 1 and Splitter 2, respectively, the pump and probe are simultaneously delivered into each of the *N* SOH-MZI based quantization channels but co-propagated in one of the arms only. After propagation inside the *N* SOH-MZI quantization channels, the probes in each of the channels are filtered by appropriate optical band-pass filters, and sent into the photodiodes and comparators for detection and binary decision.

The operation of the proposed quantizer is as follows. The pump beam consisting of pump pulses at peak power varying between 0 to *P*_max_ to be digitized are input to a 1** × ***N* splitter (Splitter 1). The splitting ratio of Splitter 1 is chosen to be 1:2:4: …:2^*N*−1^. The outputs of Splitter 1 are labeled such that the one with the weakest output power is labeled the 1-st output, the second weakest output is the 2-nd output, and so on. Thus the pump pulse peak power of the *i*-th output of Splitter 1 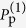 = 2^*i*−1^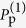, where 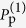 is the pump pulse peak power of the first output. When the input pulse peak power to Splitter 1 varies between 0 and *P*_max_, the pulse peak power of its *i*-th ouput varies from 0 to 2^*i*−1^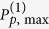, where 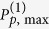 is the maximum pump pulse peak power that will enter the first SOH-MZI. The total output power of Splitter 1 is given by (2^*N*^−1)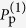 which is less than but proportional to *P*_max_. A probe beam consisting of local probe pulses at the same peak power is input into another 1** × ***N* splitter (Splitter 2). The splitting ratio of Splitter 2 is chosen to be 1:1:1: …:1.

The *N* SOH-MZIs are arranged such that the *i*-th output of Splitter 1 and an output from Splitter 2 are connected to the input of the *i*-th SOH-MZI. Thus the input pump pulse peak power to the *i*-th SOH-MZI is *P*^(i)^_p_ = 2^*i*−1^*P*^(1)^_p_. The parameters are chosen such that 2γ_sp_*P*^(1)^_p,max_*L*_eff_ = π. By this choice of parameters and from Eq. (1), when the input power to the optical quantizer varies between 0 and *P*_max_, the power transfer funcion of the *i*-th SOH-MZI will go through 2^*i*−2^ periods. In other words, the power transfer functions of the *N* SOH-MZIs will go through half-, one-, two-, …, and 2^*N*−2^ periods respectively when the sampled pulses power varies from 0 to *P*_max_. From Eq. (1), the output of all the *N* SOH-MZIs varies between 0 and *P*_s_. We use a threshold value of *P*_s_/2 in the comparator to decide the output of each SOH-MZI as 0 and 1, which depends on whether the output is larger or smaller than *P*_s_/2 or vice versa.

Figure 1(c) shows the power transfer functions of the detected probes for *N* quantization channels. By defining the decision results of b_1_ as the least significant bit (LSB) and b_*N*_ as the most significant bit (MSB), the *N*-bit Gray codes can be obtained, as shown in Fig. 1(d). For example, the code for 0 < *P* < ∆*P* is 0…000, ∆*P* < *P* < 2∆*P* is 0…001, … etc, where 0 < *P* < *P*_max_ is the input pump pulse peak power and ∆*P* = *P*_max_/2^*N*^. The proposed quantizer will quantize any pulse power in the range from 0 to *P*_max_ into a unique *N* bit binary code depending on which of the 2^*N*^ equal levels the pulse power belongs. The quantization resolution *N* equals to the number of SOH-MZI used, and the required maximum number of period is 2^*N*−2^.

### Design and modeling of the SOH horizontal slot waveguide

**Figure 2:**
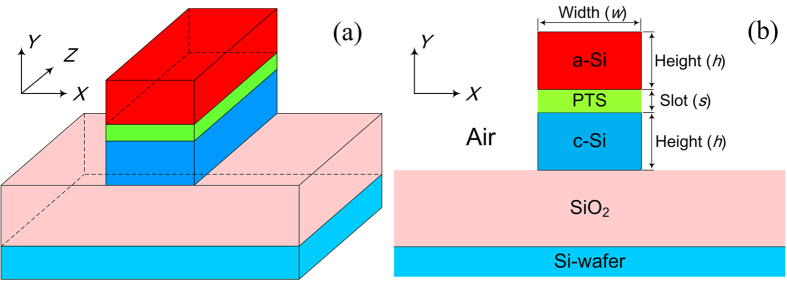
The (**a**) 3D structure and (**b**) cross-section of the SOH horizontal slot waveguide. a-Si: amorphous silicon; c-Si: crystalline silicon.

Figure 2(a,b) show the 3-D structure and cross-section of the SOH horizontal slot waveguide, respectively. A silicon dioxide layer is deposited on top of the silicon wafer to form the substrate. Above the substrate, the PTS slot layer with a thickness *s* is sandwiched between two silicon strip waveguides. The upper and lower silicon strip waveguides have the same width *w* and height *h*, which are formed by amorphous silicon (a-Si) and crystalline silicon (c-Si), respectively. The upper cladding is air. Deep-UV lithography and hard mask etching methods, together with low-pressure chemical vapor deposition (LPCVD) or plasma-enhanced chemical vapor deposition (PECVD), can be used to fabricate such a horizontal slot structure. The deposition of PTS can be achieved by spin-coating or, for a more homogeneous deposition, molecular beam deposition. The detailed fabrication procedure has been demonstrated in^26^,^28^,^33^.

**Figure 3:**
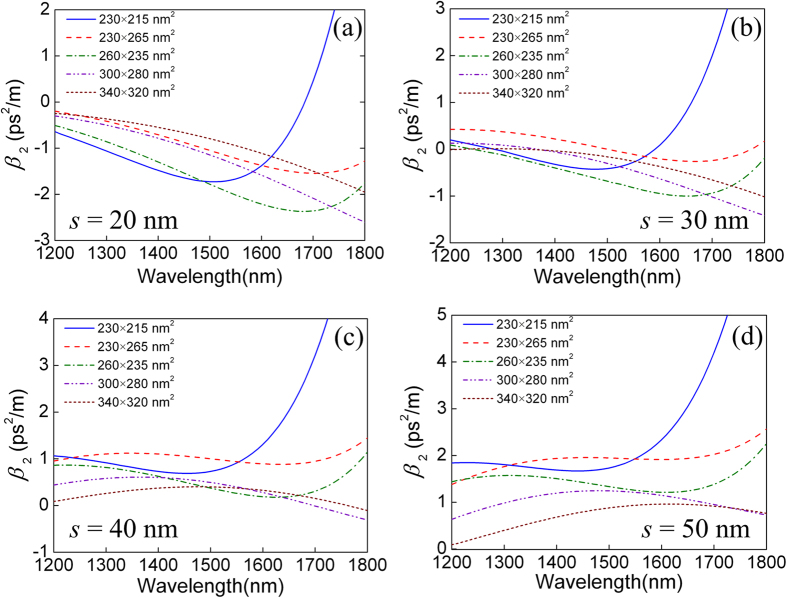
(**a–d**) show the second-order dispersion coefficients (*β*_2_) calculated as a function of wavelength when the slot thickness *s* increases from 20 to 50 nm at interval of 10 nm respectively for different combinations of waveguide dimensions *w* × *h*.

A full-vector finite element method (FEM) is used for the eigenmode calculation. The simulation domain is 4 × 4 μm^2^ with a 0.4 μm thick perfectly matched layer. An adaptive mesh refinement is used to ensure computation accuracy. Since electric field discontinuity occurs at the horizontal interface of such a waveguide structure, the quasi transverse-magnetic (TM) polarization should be concerned here. Several combinations of *w* × *h* with *w* ∈ {230 ~ 340 nm} and *h* ∈ {215 ~ 320 nm} are selected for the mode analysis. In order to obtain accurate dispersion characteristics, the material dispersions of a-Si^34^, c-Si^35^, PTS^36^, and SiO_2_^37^ are considered. Figure 3(a–d) show the second-order dispersion coefficients (*β*_2_) calculated as a function of wavelength for different waveguide dimensions when the slot thickness *s* increases from 20 to 50 nm at 10 nm interval.

**Figure 4:**
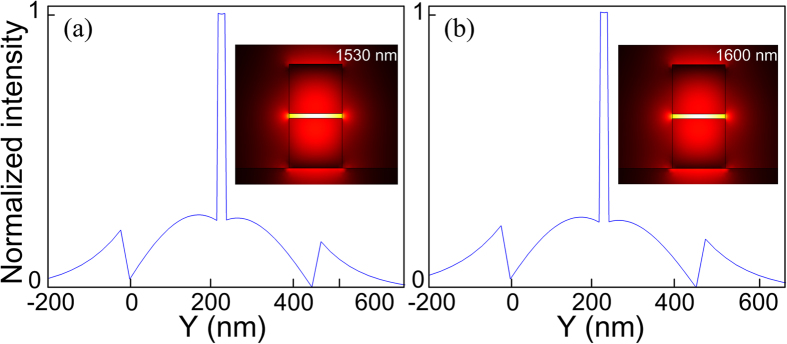
Normalized power density of the quasi-TM polarization of (**a**) probe and (**b**) pump. The insets illustrate the corresponding transverse profiles of the electric field for the quasi-TM polarization.

We observed that the *β*_2_ curves all shift towards the longer wavelength side when *w* × *h* is increased. Also, larger *s* leads to larger *β*_2_ values. For *s* = 50 nm, *β*_2_ is positive, i.e. normal dispersion, in all the wavelengths from 1200 to 1800 nm for all the combinations of *w* × *h* studied. In order to avoid parametric gain induced by FWM and soliton related processes, both the pump and probe should be located in the normal dispersion regime. Also, in order to simultaneously obtain a smaller effective area, i.e. a larger nonlinear coefficient, we chose *w* × *h* = 230 × 215 nm^2^ and *s* = 40 nm as the waveguide dimensions in the following investigation. The central wavelengths of the pump and probe are chosen to be 1600 and 1530 nm, respectively. The corresponding *β*_2_ values are 1.31 and 0.817 ps^2^/m. Figure 4(a,b) show the normalized power density of the quasi-TM polarization of probe and pump along the *Y* direction respectively. The insets are the corresponding transverse profiles of the electric field for the quasi-TM polarization. We observed that most of the electric field energy of the pump and probe is tightly confined inside the slot, which shows the excellent mode confinement ability of the proposed SOH waveguide.

**Figure 5:**
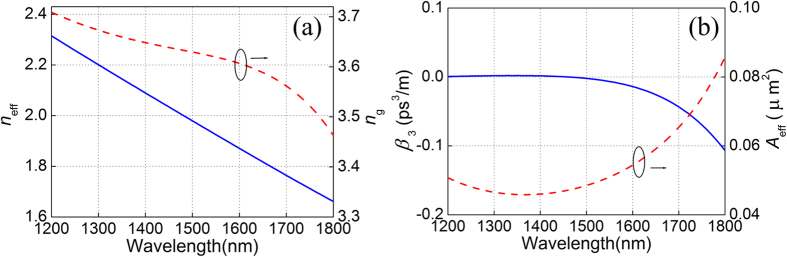
(**a**) The effective refractive index and group index, and (**b**) the third-order dispersion coefficient (*β*_3_) and effective area of the waveguide calculated as a function of wavelength.

The effective refractive index *n*_eff_ and group index *n*_g_ of the waveguide as a function of wavelength are shown in Fig. 5(a). At the wavelengths of 1530 and 1600 nm, *n*_g_ is found to be 3.625 and 3.607, respectively. Thus, the probe trails the pump with a walk-off parameter of 66.7 fs/mm. The walk-off parameter is relatively small for the waveguide so that the efficiency of XPM from pump to probe can be maintained. Figure 5(b) shows the calculated third-order dispersion coefficients (*β*_3_) and effective mode area *A*_eff_ as a function of wavelength. The *β*_3_ of the pump and probe are −0.0139 ps^3^/m and −0.0045 ps^3^/m, respectively. For large refractive index contrast of the proposed SOH waveguide, *A*_eff_ is defined by^24^,^27^


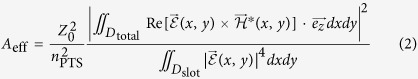


where *Z*_0_ = 377 Ω is the free space wave impedance, *n*_PTS_ the refractive index of the PTS, 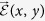 the vector electric, and 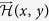 the vector magnetic field profiles of the quasi-TM polarization. The integral in the numerator covers the whole cross-section *D*_total_, whereas the integral in the denominator covers only the slot region *D*_NL_. The effective areas of the pump and probe are found to be 0.054 and 0.05 μm^2^, respectively.

The dynamics of the co-propagating pump and probe pulses inside the SOH slot waveguide can be modeled by the coupled nonlinear Schrödinger (NLS) equations as follows^37^,^38^


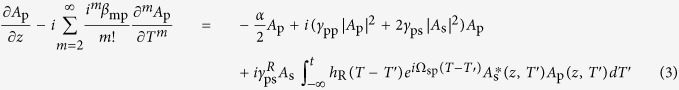



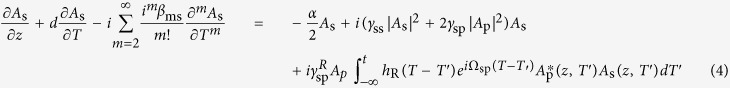


where, *T* = *t* − *z*/*v*_gp_ is the retarded frame moving with the pump at the speed of *v*_gp_, *v*_gp_ is the group velocity of pump, *A*_v_(*z*, *T*) (*v* = *p*, *s*) is the slowly varying envelope of the electric field of the pump or probe along the propagation direction *z*, *d* = (*v*_gp_ − *v*_gs_)/*v*_gp_*v*_gs_ is the walk-off parameter, *β*_mv_ is the *m*-th order dispersion coefficients for the *v* beam (*v* = *p*, *s*), α is the linear loss coefficient, and γ_uv_ (*u*, *v* = *p*, *s*) is the complex nonlinear coefficient, which is given by


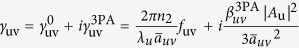


where γ_uv_^0 ^and γ_uv_^3PA^ are the real and imaginary part of the nonlinear coefficient, *β*_uv_^3PA^ is the 3PA parameter, 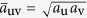 is the average effective area, and *f*_uv_ is the mode-overlap factor defined as^38^


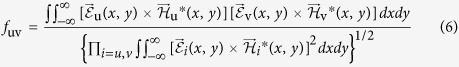


In the proposed scheme, we calculated *f*_ps_ = *f*_sp_ = 0.98. With the nonlinear refractive index *n*_2_ = 2.2 × 10^−16 ^m^2^/W^29^, the real nonlinear coefficients of pump and probe are calculated with Eq. (5) to be respectively 16,000 and 18,069 W^−1^/m, which are about 6 and 2 orders of magnitude higher than that of the HNLF^37^ and strip SOI waveguides^24^. The integral terms at the right hand sides of Eqs. (3) and (4) account for the Raman contribution. The function *h*_R_(*t*) is the Raman response function, and Ω_uv_ = *ω*_u_ − *ω*_v_ is the frequency detune. The parameter γ_uv_^*R*^ is the Raman nonlinear parameter which scales with Γ_*R*_/Ω_*R*_^38^. Here, Γ_*R*_/π is the full width at half-maximum (FWHM) of the Raman gain spectrum, and Ω_*R*_/2π is the Raman frequency shift. From the literature, the first Raman gain peak of PTS has a Raman frequency shift of 952 cm^−1^ (i.e. 28.6 THz) and an FWHM of about 310 GHz^39^,^40^. Thus, γ_uv_^*R*^ of the proposed SOH waveguide is quite small and can be neglected. Meanwhile, the 28.6 THz Raman frequency shift is much larger than the frequency difference between the pump and probe. Considering the two points above, the Raman contribution can be neglected in the coupled NLS equations. We note the frequency dependence of γ_uv_ is negligible in our models because the nonlinear refractive index and effective mode areas change slightly within the wavelength regime considered^16^.

### Quantization performances

**Figure 6:**
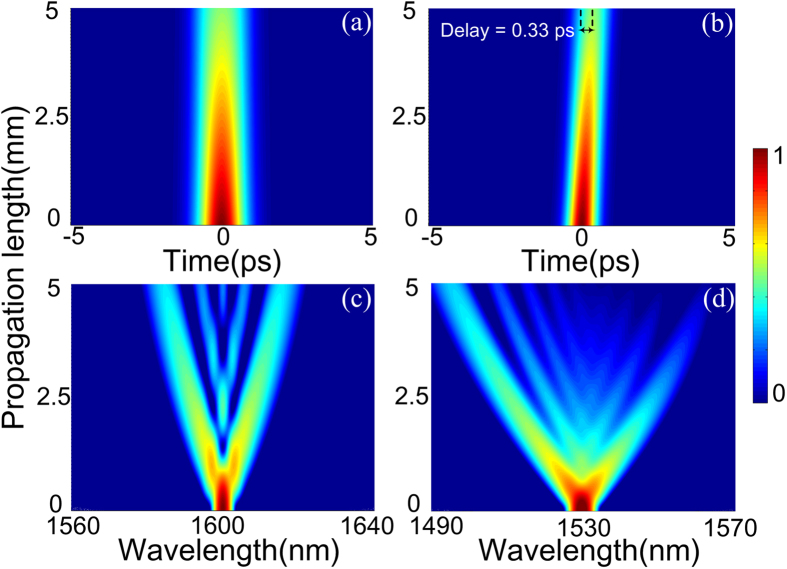
Temporal and spectral dynamic of (**a,c**) the pump pulse and (**b,d**) the probe pulse along the propagation length at *P*_p_ = 240 mW and *P*_s_ = 0.2 mW.

Eqs (3) and (4) are numerically solved by the split-step Fourier method. Gaussian pulses with 0.8 and 0.5 ps pulse width are used as the pump and probe, respectively. The pulse width of the probe is chosen to be a little smaller than that of the pump to ensure sufficient XPM interaction with negligible influence on the walk-off. The 3PA parameters of the pump and probe, i.e. *β*_pp_^3PA^ and *β*_ss_^3PA^, are 0.72 and 0.5 cm^3^/GW^2^, respectively^30^. The cross-3PA parameter is *β*_ps_^3PA^ = 0.61 cm^3^/GW^2^ from the relation *β*_ps_^3PA^ = *β*^3PA^(*ω*′), where *ω*′ = (*ω*_p_+*ω*_s_)/2 is the average angular frequency. Then, *β*_sp_^3PA^ = 0.64 cm^3^/GW^2^ can be obtained by *β*_ps_^3PA^ × (*ω*_s_/*ω*_p_)^38^. Figure 6 shows the temporal and spectral dynamics of the pump and probe along the propagation length inside the proposed SOH slot waveguide, which is 5 mm long with 4.5 dB/cm linear loss^30^,^41^. The linear loss consists of the absorption losses of the materials and the mode confinement loss of the waveguide. The pump and probe pulse peak powers are chosen to be *P*_p_ = 240 mW and *P*_s_ = 0.2 mW, respectively. It is evident that the temporal widths of both the pump (Fig. 6(a)) and probe (Fig. 6(b)) are narrowed during the propagation. We also note that the probe gradually walks off from the pump. At the end of the waveguide, the temporal separation between the pump and probe pulses is 0.33 ps. Such a temporal separation is relatively small when compared to the pulse width of the pump or probe so that the probe always overlaps with the pump during the propagation, thus maintaining efficient XPM interaction. Considering the pulse width difference between the pump and probe (0.3 ps) and the small walk-off parameter (0.33 ps), initial temporal delay between the pump and probe is no longer necessary. Figure 6(c) shows that the spectrum of the pump is almost symmetrical broadened and splits during propagation. Since the peak power of the probe pulse is much smaller than that of the pump, the spectral broadening and splitting of the pump pulse are mainly induced by SPM of the pump itself. In contrast, the spectral dynamic of the probe pulse is induced by XPM from the pump and shows typical asymmetric features, as shown in Fig. 6(d). The trailing edge of the probe pulse has a larger chirp so that the spectrum is significantly shifted towards the blue side. More important, the degree of broadening and splitting of the probe pulse is larger than that of the pump pulse because the XPM-induced chirp is about twice larger than the SPM-induced chirp.

**Figure 7:**
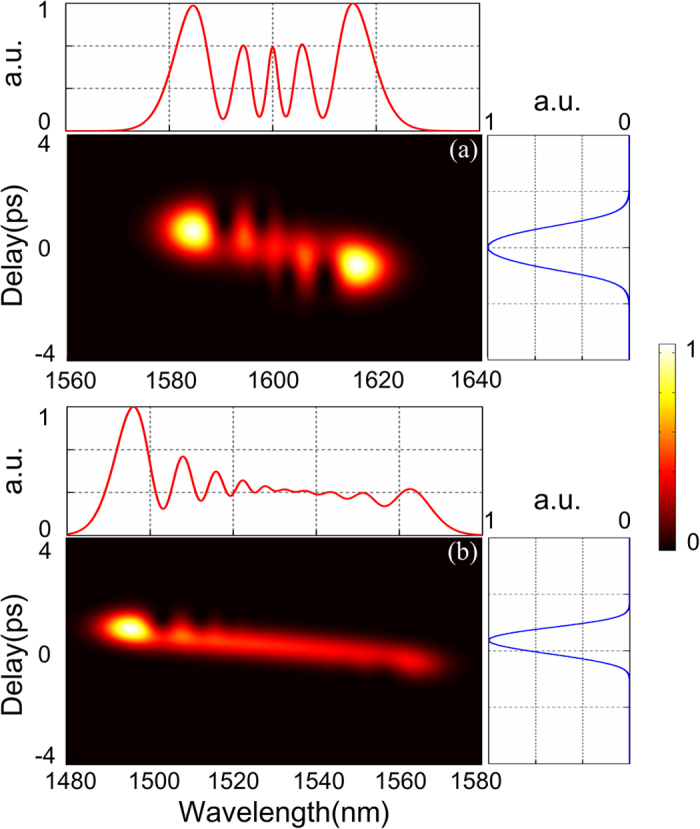
X-FROG traces of the (**a**) pump and (**b**) probe at *P*_p_ = 240 mW and *P*_s_ = 0.2 mW, respectively. The corresponding normalized temporal (blue line) and spectral (red line) profiles are also illustrated.

In order to simultaneously show the temporal and spectral dynamics, the cross-correlation frequency-resolved optical gating (X-FROG) traces of the pump and probe at *P*_p_ = 240 mW and *P*_s_ = 0.2 mW are illustrated in Fig. 7(a,b), respectively. We observed that the spectra of the pump and probe split into 5 and 10 peaks, respectively. By estimating the FWHM, the pump spectrum is both red- and blue-shifted by 20 nm to 1620 and 1580 nm respectively from the central wavelength of 1600 nm. The probe gets a larger blue-shift of 41 nm to about 1489 nm and a red-shift of 38 nm to 1568 nm from the central wavelength of 1530 nm. Most of the spectral energy of probe is contained in the blue-shifted component around 1495 nm. This wavelength can be used as the central wavelength of the optical bandpass filter (OBPF) to extract the probe. Unlike the varying spectra, the temporal shapes of the pump and probe pulses remain almost unchange with only slight compression. Thus the XPM effect during propagation inside and the interference at the end of the SOH-MZI are not affected. The temporal shape of pulse is mainly affected by the dispersion effect. The dispersion length of pump and probe pulses can be calculated by *L*_Dv_ = *T*_0v_^2^/|*β*_2v_|, (*v* = *p*, *s*). The dispersion lengths of the pump *L*_Dp_ = 0.49 m and that of the probe *L*_Ds_ = 0.31 m are both much larger than the waveguide length of 5 mm, which means dispersion does not significantly affect the pulse dynamic. In contrast, the nonlinear lengths are given by *L*_NLuv_ = 1/γ_vu_*P*_u_, (*u*, *v* = *p*, *s*). The SPM length of the pump is *L*_NLpp_ = 0.26 mm and that of the probe is *L*_NLss_ = 0.28 m. The XPM lengths of pump to probe and probe to pump are *L*_NLps_ = 0.24 mm and *L*_NLsp_ = 0.3 m, respectively. The nonlinear lengths indicate that the SPM and XPM contribution of the probe is negligible. Thus, the dynamic of the co-propagating pulses is dominated by the nonlinear contribution of pump to the probe.

**Figure 8:**
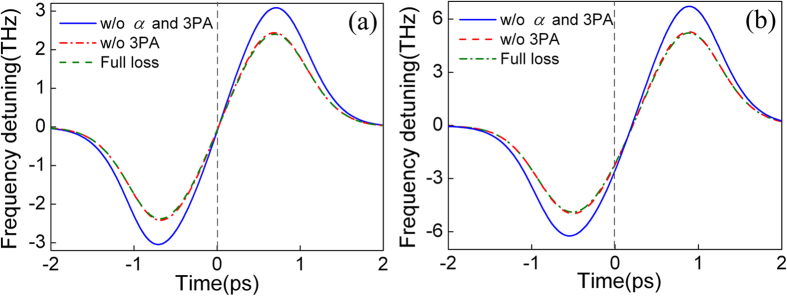
Frequency detunes of the (**a**) pump and (**b**) probe under different loss conditions.

To analyze the influence of linear loss α and 3PA, the frequency detunes of the pump and probe are calculated by taking the derivative of the phase at different loss conditions, as shown in Fig. 8(a,b). The leading and trailing edges of the pump pulse have almost the same chirp. The maximum and minimum frequency detunes of the pump are 2.39 and −2.38 THz respectively with both linear loss and 3PA. However, in the case of the probe, the trailing edges have a larger chirp, and the frequency detune is asymmetric. The results agree well with that shown in Figs 6 and 7. With both linear loss and 3PA, the maximum and minimum frequency detunes of the probe are 5.23 and −4.92 THz, respectively. Figure 8 also shows that 3PA has little effect on the frequency detune. Taking the probe as example, the maximum and minimum frequency detunes can reach 5.27 and −4.96 THz respectively without 3PA. But, when both linear loss and 3PA are ignored, the maximum and minimum frequency detunes increase to 6.72 and −6.23 THz, respectively.

**Figure 9:**
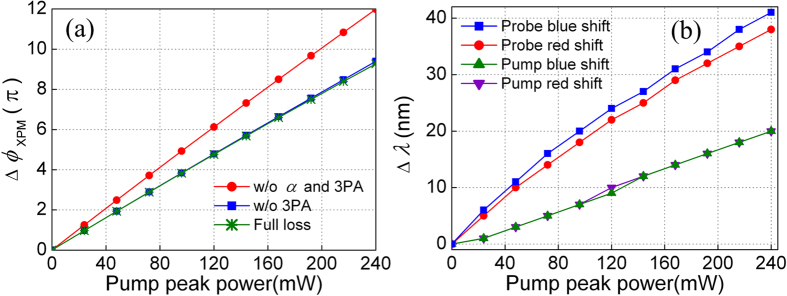
(**a**) The maximum XPM-induced phase shift ∆*ϕ*_XPM_ as a function of *P*_p_ under different loss conditions, and (**b**) the wavelength broadening ∆λ of the pump and probe as a function of *P*_p_ when both linear loss and 3PA are included.

Figure 9(a) shows the maximum XPM-induced phase shifts ∆*ϕ*_XPM_ when *P*_p_ increases from 0 to 240 mW under different loss conditions. The ∆*ϕ*_XPM_ is obtained by calculating the phase change of the probe pulse before and after propagation inside the waveguide with Eqs (3) and (4). We observe the ∆*ϕ*_XPM_ varies almost linearly with the pump pulse peak power without linear loss and 3PA. At the pump pulse peak power of 240 mW, ∆*ϕ*_XPM_ reaches about 12π. When only linear loss or both linear loss and 3PA are considered, ∆*ϕ*_XPM_ also varies almost linearly with the pump pulse peak power but at a lower rate. Even with both linear loss and 3PA, a π phase shift can be achieved by only 24 mW pump pulse peak power. The phase shifts ∆*ϕ*_XPM_ reaches 9.3π at *P*_p_ = 240 mW. The relation ∆*ϕ* ≈ (*K* − 0.5)/π can be used to estimate the amount of nonlinear phase shift, where K is the number of peaks of the output spectrum^16^. For the 9.3π phase shift, the peak number of probe spectrum should be 10, which is quite consistent with the spectral results shown in Figs 6(d) and 7(b). The maximum phase shift of pump at 240 mW is calculated to be 4.5π, which also agrees well with the 5 spectrum peaks result in Figs 6(c) and 7(a). Figure 9(b) shows the wavelength broadening ∆λ of the pump and probe as a function of *P*_p_ when both linear loss and 3PA are included. It can be seen that ∆λ of both the pump and probe pulses increase monotonically with the pump pulse peak power. The frequency detune of the probe is roughly twice that of the pump. When the pump pulse peak power is 240 mW, the maximum ∆λ is found to be 41 nm, which is mainly contributed by the blue shift of probe.

**Figure 10:**
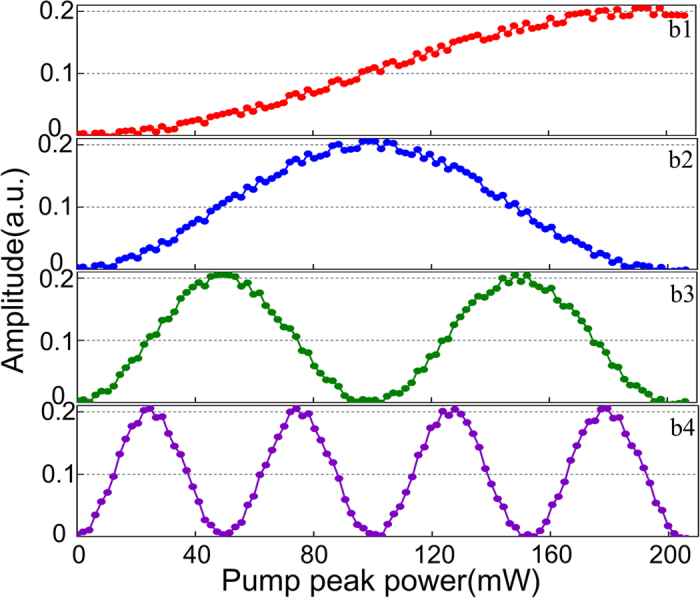
Output waveforms of the four quantization channels b1, b2, b3, and b4.

After XPM and interference in the SOH-MZI, the probe is filtered out and detected by the PIN. As an illustration, we used four SOH-MZIs labeled as b_1_  ~  b_4_ as the parallel quantization channels for 4-bit resolution. The maximum pump pulse peak power entering the SOH waveguides of the MSB channel (b_4_) is set to be 206 mW, which corresponds to ∆*ϕ*_XPM_ = 8π, i.e. four-periods for the power transfer function of SOH-MZI. By using a splitter with splitting ratio of 1:2:4:8, the maximum pump pulse peak powers of b_1_, b_2_, b_3_, and b_4_ are 25.75, 51.5, 103, and 206 mW, respectively. The corresponding nonlinear lengths of the four channels are 2.2, 1.1, 0.56, and 0.28 mm, respectively, which are all less than the waveguide length of 5 mm. Accordingly, the ∆*ϕ*_XPM_ of b_1_, b_2_, b_3_, and b_4_ channels are π, 2π, 4π, and 8π which correspond to the periods of half-, one-, two-, and four-, respectively. Thus, the four power transfer functions with successively doubled period can be achieved. Since the ∆*ϕ*_XPM_ does not vary straightly linearly with the pump pulse peak power, some phase errors would be generated, which would lead to non-integer period and quantization errors in the decision results. However, since the degree of non-linearity between the ∆*ϕ*_XPM_ and pump pulse peak power is very small, the phase errors are predicted not to significantly degrade the quantization performance. Furthermore, we assume the PIN noise to be the typical 10 pA/(Hz)^−1/2^ thermal noise and a dark current of 20 pA in the simulations. Figure 10 shows the output waveforms of the four quantization channels. The amplitude of each channel fluctuates because of the PIN noise.

**Figure 11:**
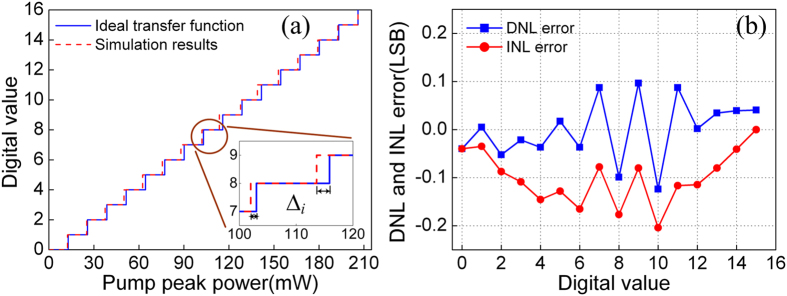
(**a**) Quantization transfer function of the proposed 4-bit quantizer. Inset: the zoom-in view, and (**b**) differential nonlinear (DNL) and integral nonlinear (INL) errors.

By making decision at half-amplitude, combinations of the binary codes of the four parallel channels are used to obtain the quantization transfer function, as shown in Fig. 11(a). We observed that the difference between the ideal and the simulated quantization transfer function is very small. The inset shows the zoom-in view of one of the quantization stages, in which the difference ∆_*i*_ can be seen clearly. Figure 11(b) shows the differential nonlinear (DNL) and integral nonlinear (INL) errors calculated with the simulated quantization transfer function. The maximum DNL and INL errors are found to be only 0.12 and 0.2 LSB, respectively. The signal-to-noise ratio (SNR) and effective number of bit (ENOB) are estimated to be 23.42 dB and 3.89-bit, respectively, using the results illustrated in Fig. 11. We note that only 0.11-bit difference is generated compared to the ideal resolution.

## Discussion

For practical applications of third-order nonlinear effects based silicon functional devices, the coupling loss is a crucial issue. The primary criterion for the proposed quantization scheme to be technically feasible is that the pump pulse peak power after coupling into the SOH waveguide could reach 206 mW. The optical components before the SOH waveguide, namely the MMI, the multiplexer, the strip-to-slot converter, and the access strip waveguides, will have coupling and propagation losses. From the literature^32^, the losses induced by the MMI, strip-to-slot converter, and access strip waveguides are 0.2, 0.02, and 1 dB, respectively. The coupling loss of the multiplexer can be assumed to be 0.28 dB as reported in^42^. Since the footprint is only 1.2 × 2 μm^2^, the propagation loss of the multiplexer can be neglected. The coupling of the pump into the MMI can be realized by the inverted taper coupling technique, with which a low coupling loss of 0.36 dB is obtained experimentally^43^. Therefore, the total loss from the input of the quantizer to the front end of the SOH waveguide could be only 1.86 dB. Thus the input pump pulse peak power should be 316 mW. Assuming a pulse repetition rate of 40 GHz, the average power is only 10.1 mW, which is achievable with current mode-locked laser technologies. Even using the common grating couplers for the fiber-to-waveguide coupling, which allows for on-wafer testing, the coupling efficiency of 20% (7 dB loss) has been achieved experimentally in^44^. Under this condition, the total loss becomes 8.5 dB so that the input pump pulse peak power should be 1.46 W. The corresponding average power should be 46.7 mW, which is also feasible. It should be noted that the coupling losses have no influence on the probe since its initial peak power is far less than the pump. Moreover, the optical sampling module can be realized by using the SOI-based optical parametric sampling technique and integrated on a separate chip^45^,^46^. From the above analysis, coupling loss is unlikely to affect negatively the feasibility of the proposed scheme because of the low pump pulse peak power threshold we obtained and the current efficient waveguide coupling techniques. The proposed quantizer can be applied in the cascade optical quantization scheme, which cascades two optical quantizer with lower resolution to achieve remarkable resolution enhancement^12^,^47^. For instance, an 8-bit resolution was achieved in^12^ with only 8-periods power transfer function. The proposed quantizer can achieve 8-periods by increasing the pump pulse peak power to 412 mW, which is feasible in practice.

In summary, we propose an on-chip integratable all-optical quantizer using the strong XPM effect in an SOH horizontal slot waveguide filled with PTS. By carefully designing the dimensions of the SOH waveguide, tight sub-wavelength confinement of the pump and probe in the 40 nm slot layer is achieved, together with large nonlinear coefficients up to 16,000 and 18,069 W^−1^/m for the pump and probe, respectively. A low walk-off parameter of 66.7 fs/mm is also obtained, which generates only 0.33 ps delay between the pump and probe pulses in a 5 mm long waveguide. Such a small delay enables the probe pulse to always overlap with the pump pulse during propagation and maintain efficient XPM interaction. In the simulation, we show that only 206 mW pump pulse peak power is required for an 8π phase shift of the probe, which corresponds to a 4-bit resolution with ENOB of 3.89-bit. The low peak power threshold renders the proposed quantization scheme feasible even taking into account practical coupling loss and ensures the interconnectivity with other optical functional devices. It is also believed that the quantizer can be combined with cascade optical quantization scheme to achieve a higher resolution. The proposed quantizer is expected to have significant applications in photonic integratable optical communication links, optical interconnection networks, and real-time signal processing systems.

## Methods

### Model of the X-FROG trace

The X-FROG trace obtained in our simulation is based on the difference-frequency generation XFROG algorithm, which is given by^37^


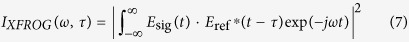


where *E*_sig_(*t*) is the electric field of the measured signal, *E*_ref_^*^(*t* − τ) is the conjugate electric field of the reference signal delayed by time τ. The reference signal is characterized by the initial input signal.

### Estimation of the ENOB

Assuming that the shape of analog input for digitization is arbitrary, and the amplitude obeys uniform distribution. The SNR and ENOB can be estimated by^1^,^11^


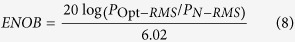



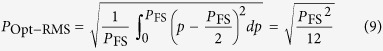



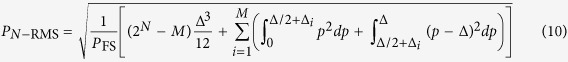


where *P*_Opt-RMS_ and *P*_N-RMS_ are the root-mean-square of the optical power and the quantization errors respectively, *P*_FS_ is the full-scale optical power range, ∆ = *P*_FS_/2^*N*^ is the power of the LSB, *N* is the ideal quantization resolution, ∆_*i*_ is the nonlinear error of the *i*-th quantization stage, and *M* is the number of quantization stages, which have the nonlinear errors.

## Additional Information

**How to cite this article**: Kang, Z. *et al.* On-chip integratable all-optical quantizer using strong cross-phase modulation in a silicon-organic hybrid slot waveguide. *Sci. Rep.* **6**, 19528; doi: 10.1038/srep19528 (2016).

## References

[b1] Walden R. H. Analog-to-digital converter survey and analysis. IEEE J. Sel. Areas Commun. 17, 539–550 (1999).

[b2] Valley G. C. Photonic analog-to-digital converters. Opt. Express 15, 1955–1982 (2007). 19532436 10.1364/oe.15.001955

[b3] Khilo A. *et al.* Photonic ADC: overcoming the bottleneck of electronic jitter. Opt. Express 20, 4454–4469 (2012). 22418205 10.1364/OE.20.004454

[b4] Sarantos C. H. & Dagli N. A photonic analog-to-digital converter based on an unbalanced Mach-Zehnder quantizer. Opt. Express 18, 14598–14603 (2010). 20639945 10.1364/OE.18.014598

[b5] Wang Y., Zhang H. M., Wu Q. W. & Yao M. Y. Improvement of photonic ADC based on phase-shifted optical quantization by using additional modulators. IEEE Photon. Technol. Lett. 24, 566–568 (2012).

[b6] Oda S. & Maruta A. A novel quantization scheme by slicing supercontinuum spectrum for all-optical analog-to-digital conversion. IEEE Photon. Technol. Lett. 17, 465–467 (2005).

[b7] Nishitani T., Konishi T. & Itoh K. Resolution improvement of all-optical analog-to-digital conversion employing self-frequency shift and self-phase-modulation-induced spectral compression. IEEE J. Sel. Topics Quantum Electron. 14, 724–732 (2008).

[b8] Satoh T., Takahashi K., Matsui H., Itoh K. & Konishi T. 10-GS/s 5-bit real-time optical quantization for photonic analog-to-digital conversion. IEEE Photon. Technol. Lett. 24, 830–832 (2012).

[b9] Takahashi K., Matsui H., Nagashima T. & Konishi T. Resolution upgrade toward 6-bit optical quantization using power-to-wavelength conversion for photonic analog-to-digital conversion. Opt. Lett. 38, 4864–4867 (2013). 24322152 10.1364/OL.38.004864

[b10] Ikeda K. *et al.* Design considerations of all-optical A/D conversion: nonlinear fiber-optic Sagnac-loop interferometer-based optical quantizing and coding. J. Lightwave Technol. 24, 2618–2628 (2006).

[b11] Miyoshi Y., Takagi S., Namiki S. & Kitayama K. I. Multiperiod PM-NOLM with dynamic counter-propagating effects compensation for 5-bit all-optical analog-to-digital conversion and its performance evaluations. J. Lightwave Technol. 28, 415–422 (2010).

[b12] Miyoshi Y., Namiki S. & Kitayama K. I. Performance evaluation of resolution-enhanced ADC using optical multiperiod transfer functions of NOLMs. IEEE J. Sel. Topics Quantum Electron. 18, 779–784 (2012).

[b13] Reed G. T. The optical age of silicon. Nature 427, 595–596 (2004). 10.1038/427595b 14961105

[b14] Almeida V. R., Barrios C. A., Panepucci R. R. & Lipson M. All-optical control of light on a silicon chip. Nature 431, 1081–1084 (2004). 15510144 10.1038/nature02921

[b15] Kuo Y. H. *et al.* Demonstration of wavelength conversion at 40 Gb/s data rate in silicon waveguides. Opt. Express 14, 11721–11726 (2006). 19529594 10.1364/oe.14.011721

[b16] Dadap J. I. *et al.* Nonlinear-optical phase modification in dispersion-engineered Si photonic wires. Opt. Express 16, 1280–1299 (2008). 18542202 10.1364/oe.16.001280

[b17] Matsuda N. *et al.* All-optical phase modulations in a silicon wire waveguide at ultralow light levels. Appl. Phys. Lett. 95, 171110 (2009).

[b18] Boyraz O., Koonath P., Raghunathan V. & Jalali B. All optical switching and continuum generation in silicon waveguides. Opt. Express 12, 4094–4102 (2004). 19483951 10.1364/opex.12.004094

[b19] Leuthold J., Koos C. & Freude W. Nonlinear silicon photonics. Nat. photon. 4, 535–544 (2010).

[b20] Dekker R. *et al.* Ultrafast Kerr-induced all-optical wavelength conversion in silicon waveguides using 1.55 μm femtosecond pulses. Opt. Express 14, 8336–8346 (2006). 19529210 10.1364/oe.14.008336

[b21] Astar W. *et al.* Tunable wavelength conversion by XPM in a silicon nanowire, and the potential for XPM-multicasting. J. Lightwave Technol. 28, 2499–2511 (2010).

[b22] Astar W. *et al.* Conversion of 10 Gb/s NRZ-OOK to RZ-OOK utilizing XPM in a Si nanowire. Opt. Express 17, 12987–12999 (2009). 19654703 10.1364/oe.17.012987

[b23] Astar W. *et al.* All-optical format conversion of NRZ-OOK to RZ-OOK in a silicon nanowire utilizing either XPM or FWM and resulting in a receiver sensitivity gain of 2.5 dB. IEEE J. Sel. Topics Quantum Electron. 16, 234–249 (2010).

[b24] Koos C., Jacome L., Poulton C., Leuthold J. & Freude W. Nonlinear silicon-on-insulator waveguides for all-optical signal processing. Opt. Express 15, 5976–5990 (2007). 19546900 10.1364/oe.15.005976

[b25] Jones T. B. & Hochberg M. Silicon photonics slot machine. Nat. photon. 3, 193–194 (2009).

[b26] Koos C. *et al.* All-optical high-speed signal processing with silicon-organic hybrid slot waveguides. Nat. Photon. 3, 216–219 (2009).

[b27] Muellner P., Wellenzohn M. & Hainberger R. Nonlinearity of optimized silicon photonic slot waveguides. Opt. Express 17, 9282–9287 (2009). 19466180 10.1364/oe.17.009282

[b28] Martinez A. *et al.* Ultrafast all-optical switching in a silicon-nanocrystal-based silicon slot waveguide at telecom wavelengths. Nano Lett. 10, 1506–1511 (2010). 20356059 10.1021/nl9041017

[b29] Lawrence B. L. *et al.* Large purely refractive nonlinear index of single crystal P = toluene sulphonate (PTS) at 1600 nm. Electron. Lett. 30, 447–448 (1994).

[b30] Polyakov S., Yoshino F., Liu M. G. & Stegeman G. Nonlinear refraction and multiphoton absorption in polydiacetylenes from 1200 to 2200 nm. Phys. Rev. B 69, 115421 (2004).

[b31] Palmer R. *et al.* Silicon-organic hybrid MZI modulator generating OOK, BPSK and 8-ASK signals for up to 84 Gbit/s. IEEE Photon. J. 5, 6600907 (2013).

[b32] Wooten E. L., Stone R. L., Miles E. W. & Bradley E. M. Rapidly tunable narrowband wavelength filter using LiNbO_3_ unbalanced Mach-Zehnder interferometers. J. Lightwave Technol. 14, 2530–2536 (1996).

[b33] Jordana E. *et al.* Deep-UV lithography fabrication of slot waveguides and sandwiched waveguides for nonlinear applications. *IEEE International Conference on Group IV Photonics*, (Institute of Electrical and Electronics Engineers 2007), paper WB4.

[b34] Polyanskiy M. N. Refractive index database., (2008) Date of access: 30/02/2084. http://refractiveindex.info.

[b35] Yin L. H., Lin Q. & Agrawal G. P. Soliton fission and supercontinuum generation in silicon waveguides. Opt. Lett. 32, 391–393 (2007). 17356663 10.1364/ol.32.000391

[b36] Bhowmik A. K. & Thakur M. Self-phase modulation in polydiacetylene single crystal measured at 720–1064 nm. Opt. Lett. 26, 902–904 (2001). 18040486 10.1364/ol.26.000902

[b37] Agrawal G. P. Nonlinear Fiber Optics - the 4th edition (Academic Press, San Diego, 2001).

[b38] Lin Q., Painter O. J. & Agrawal G. P. Nonlinear optical phenomena in silicon waveguides: Modeling and applications. Opt. Express 15, 16604–16644 (2007). 19550949 10.1364/oe.15.016604

[b39] Batchelder D. N. & Bloor D. An investigation of the electronic excited state of a polydiacetylene by resonance Raman spectroscopy. J. Phys. C: Solid State Phys. 15, 3005–3018 (1982).

[b40] Iqbal Z., Chance R. R. & Baughman R. H. Electronic structure change at a phase transition in a polydiacetylene crystal. J. Chem. Phys. 66, 5520–5525 (1977).

[b41] An L. L., Liu H. J., Sun Q. B., Huang N. & Wang Z. L. Wavelength conversion in highly nonlinear silicon-organic hybrid slot waveguides. Appl. Opt. 53, 4886–4893 (2014). 25090318 10.1364/AO.53.004886

[b42] Zhang Y. *et al.* A compact and low loss Y -junction for submicron silicon waveguide. Opt. Express 21, 1310–1316 (2013). 23389024 10.1364/OE.21.001310

[b43] Pu M. H., Liu L., Ou H. Y., Yvind K. & Hvam J. M. Ultra-low-loss inverted taper coupler for silicon-on-insulator ridge waveguide. Opt. Commun. 283, 3678–3682 (2010).

[b44] Galan J. V. *et al.* Silicon sandwiched slot waveguide grating couplers. Electron. Lett. 45, 20093138 (2009).

[b45] Salem R. *et al.* High-speed optical sampling using a silicon-chip temporal magnifier. Opt. Express 17, 4324–4329 (2009). 19293857 10.1364/oe.17.004324

[b46] Foster M. A. *et al.* Broad-band optical parametric gain on a silicon photonic chip. Nat. Photon. 441, 960–963 (2006). 10.1038/nature04932 16791190

[b47] Kang Z. *et al.* Resolution-enhanced all-optical analog-to-digital converter employing cascade optical quantization operation. Opt. Express 22, 21441–21453 (2014). 25321522 10.1364/OE.22.021441

